# A Mid-Cretaceous Origin of Sociality in Xylocopine Bees with Only Two Origins of True Worker Castes Indicates Severe Barriers to Eusociality

**DOI:** 10.1371/journal.pone.0034690

**Published:** 2012-04-12

**Authors:** Sandra M. Rehan, Remko Leys, Michael P. Schwarz

**Affiliations:** 1 School of Biological Sciences, Flinders University of South Australia, Adelaide, South Australia, Australia; 2 Department of Biological Sciences, Brock University, St. Catharines, Ontario, Canada; 3 Evolutionary Biology Unit, South Australia Museum, Adelaide, South Australia, Australia; Arizona State University, United States of America

## Abstract

The origin of sterile worker castes, resulting in eusociality, represents one of the major evolutionary transitions in the history of life. Understanding how eusociality has evolved is therefore an important issue for understanding life on earth. Here we show that in the large bee subfamily Xylocopinae, a simple form of sociality was present in the ancestral lineage and there have been at least four reversions to purely solitary nesting. The ancestral form of sociality did not involve morphological worker castes and maximum colony sizes were very small. True worker castes, entailing a life-time commitment to non-reproductive roles, have evolved only twice, and only one of these resulted in discrete queen-worker morphologies. Our results indicate extremely high barriers to the evolution of eusociality. Its origins are likely to have required very unusual life-history and ecological circumstances, rather than the amount of time that selection can operate on more simple forms of sociality.

## Introduction

The evolution of life has been marked by a number of major but very infrequent transitions, such as the origins of eukaryotes, multicellularity, and sexual reproduction [1]. One of these key transitions is the origin of eusociality, where individuals within groups show life-time specialization in reproductive or non-reproductive roles. Like the other transitions, eusociality has arisen very rarely, but where it has arisen it has had a major impact in shaping biotic and ecosystem diversity.

The question of why eusociality has been so successful, yet its origins have been so rare, has been a major puzzle in evolutionary biology. One approach to this issue has involved identifying so-called ‘pre-adaptations’ or ‘conditions’ for eusociality – combinations of genetic, life-history and ecological features that facilitate the evolution of strong forms of altruism [2]–[5]. We would expect, *a priori*, that such conditions should be very restrictive, otherwise transitions to eusociality would be common. Past attempts at identifying these conditions have looked for common factors underlying eusocial origins, but this approach runs the risk of casting a net too broadly. Common factors might be identifiable, but whether or not they are sufficient is less straightforward.

One way to address this problem of identifying sufficient factors is to examine clades where putative pre-adaptations have been in place for long periods of evolutionary time and ask whether they have facilitated transitions to eusociality. For this, we require taxa where the history of conditions or pre-adaptations for eusociality are known, and where the origins of eusociality can be identified.

The bees contain multiple origins of eusociality, but most bee species are solitary or only weakly social. Molecular studies have indicated three origins of eusociality in halictine bees with up to 12 subsequent losses [6], and a single origin in the corbiculate bees, comprising the tribes Bombini, Euglossini, Meliponini and the Apini, with one subsequent loss [7]. Although tribal relationships for the corbiculates have not been firmly resolved for a long time [8], two recent molecular studies [9], [10] using seven and 12 nuclear genes respectively both recover the same (Bombini+Meliponini)+(Apini+Euglossini) phylogeny that was used to infer the single origin of sociality by [7].

The only other bee group where eusociality has been reported is the subfamily Xylocopinae, which is in the same family Apidae as the corbiculates. The Xylocopinae comprises four extant tribes. The relictual tribe Manueliini contains three species and two of these are known to be solitary [11], [12]. The tribes Ceratinini and Xylocopini contain both solitary and social species, but sociality in these groups never entails lifelong castes. Instead, it involves reproductive hierarchies among totipotent females, where the reproductive status of nestmates can change over time and where sterile worker castes are not evident [13]–[20]. Lastly, all species in the tribe Allodapini exhibit at least weak forms of sociality [21], but many species have well-defined behavioural castes [22] and in two species, *Exoneurella tridentata* and *Hasinamelissa minuta*, queen and worker castes are discrete and life-long [23], [24].

The widespread distribution of sociality in the Xylocopinae could reflect multiple origins of sociality, or else a single and older origin followed by both reversions to solitary behaviour and elaborations into complex sociality. Both possibilities have major consequence for understanding social evolution. Repeated origins of sociality would suggest the presence of some facilitating factor or pre-adaptation common to the subfamily, whereas a single origin would entail a longer history of social behaviour, but one where there have been very few elaborations into complex sociality. An ancient origin of weak sociality in the Xylocopinae followed by very few origins of complex sociality would indicate very formidable barriers to highly eusocial behaviour even when simple forms of sociality are in place.

**Figure 1:**
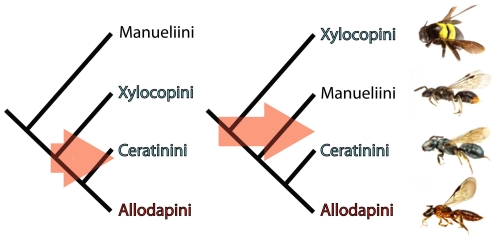
Alternative scenarios of carpenter bee relationships with likely implications for origins of sociality. Studies conflict over whether Manueliini or Xylocopini is the most basal tribe the Xylocopinae. All allodapines are social and most ceratinines and xylocopines are social, whereas the only two well-studied species of *Manuelia* are solitary. A Manueliini-basal phylogeny (left) would make it more likely that sociality is not the ancestral state for the subfamily as well as imply a more recent origin of sociality. Arrows contrast the possible ranges in timings of social origins, but more than one origin is still possible under both scenarios.

Distinguishing between a single and multiple origins of sociality in the Xylocopinae requires that phylogenetic relationships of the constituent tribes are well resolved. Previous studies [7], [25]–[28] have all recovered Allodapini and Ceratinini as distally-placed sister tribes, but disagreed on whether the most basal tribe is Manueliini or Xylocopini. The phylogenetic positions of these two latter tribes is critical because the two Manueliini species that have been studied in detail are solitary [11], [12], [29], whereas more than half of the studied Xylocopini species are social [19], [30]. A basal position for Manueliini is therefore likely to decrease the likelihood that sociality is ancestral for the Xylocopinae as a whole (Figure 1). Here we use phylogenetic analyses based on two mitochondrial and two nuclear genes from 70 Xylocopinae species to produce a well resolved and strongly supported phylogeny of the xylocopine tribes. We then use social data from studies of 47 Xylocopinae species to infer a single origin of sociality in this subfamily and some traits of the ancestral social lineage.

## Results

**Figure 2:**
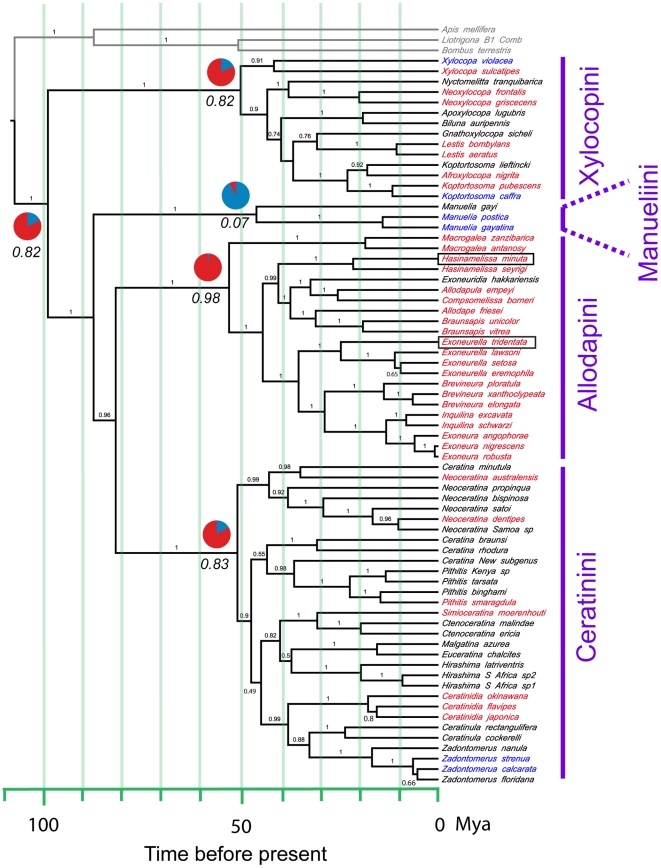
Evolution of sociality in the Xylocopinae. Chronogram of the Xylocopinae based on 70 species from all extant xylocopine tribes. The chronogram was derived from a log normal relaxed clock model in BEAST and posterior probability support for each node is indicated by numbers above branches. Social species are coloured red, solitary species are blue and species where social status are unknown are black. Outgroup clades are indicated by grey branches and the root node, uniting the outgroup and the Xylocopinae, was set at 107 Mya. The two Xylocopinae species known to have true worker castes are indicated by black rectangles. The relative probabilities of social and solitary as states for key internal nodes were estimated using a Bayesian analysis and are summarized as pie charts with the probability of being social (red slices) indicated by italic numbers. For Xylocopini and Ceratinini, which are both monogeneric, we have used the subgeneric rather than generic names.

We used three different Bayesian approaches and a maximum parsimony analysis to recover the phylogeny of the Xylocopinae. All analyses indicated that the Xylocopini comprise the most basal tribe and Manueliini was the next-most basal tribe (Figure 2 and Figures S1, S2, S3 electronic supplementary material). Bayesian posterior probability support for these bifurcations, from each of the three analytic approaches, was ≥98% for all of the tribal and supra-tribal nodes, though MP bootstrap support was lower. A Bayes Factor test, comparing log likelihoods for an unconstrained analysis with those for an analysis where Manueliini was constrained to a basal position in the subfamily, indicated very strong support (BF = 24.170) for Xylocopini as the most basal tribe.

We developed chronograms for the Xylocopinae using an uncorrelated log normal relaxed clock model, setting the root node uniting the corbiculate apid bees with the Xylocopinae at 107 Mya, based on a recent molecular phylogenetic analysis of the Apidae [7]. Using this calibration date we found the crown age of the Xylcopinae to be in the mid-Cretaceous, about 98 Mya, with the crown ages of Manueliini and Xylocopini dated at ca. 46 and 50 My, and Allodapini and Ceratinini at ca. 53 and 51 Mya respectively (Table 1). We also varied the set age of the root node to 120, 100 and 90 Mya to explore the effect of uncertainty in age of this node (Table 1). The lower value of 90 Mya gave a crown age for the Xylocopinae of ca. 83 Mya, still well in the Cretaceous. Our tribal crown ages based on a root node age of 107 Mya correspond well with other molecular studies that have included these tribes [7], [31]–[34].

**Table 1:** Age estimates of Xylocopinae root age and tribe origins obtained from a relaxed clock model.

Root node set to:	90 My	100 My	107 My	120 My
Xylcopinae	82.91	92.12	98.57 (103.30)	110.54
M+C+A	73.23	81.36	87.06 (90.70)	97.63
C+A	68.52	76.14	81.47 (85.46)	91.36
Manueliini (M)	38.65	42.94	45.95 (54.42)	51.53
Xylocopini (X)	42.01	46.68	49.95 (61.90)	56.02
Allodapini (A)	44.46	49.40	52.86 (49.18)	59.28
Ceratinini (C)	42.68	47.42	50.74 (50.11)	56.91
MRCA	37.30	41.44	44.34 (41.59)	49.73

The root node, connecting the corbiculate outgroup with the Xylocopinae, was set at four different values, ranging from 90 Mya to 120 Mya to explore the effects on internal node estimates. The root node age set to 107 Mya corresponds to the estimate by Cardinal and Danforth [7]. Age estimates are also given for the node uniting Manueliini, Ceratinini and Allodapini (M+C+A), the node uniting the Ceratinini and Allodapini (C+A), and the most recent common ancestor (MRCA) for the two allodapine species (*Exoneurella tridentata* and *Hasinamelissa minuta*) that have true worker castes. Bayesian analyses indicate that this MRCA did not have true castes, so that the age of this node predates the two origins of true workers. Node age estimates are also given in parentheses for a penalized likelihood transformation of the consensus phylogram obtained from a MrBayes analysis where the root node was set to 107 Mya.

Importantly, our penalized likelihood estimation of divergence dates, based on a MrBayes phylogram, gave very similar results to our BEAST analysis (Table S1 and Figure S1). When the root node was set to 107 Mya for both analyses, age discrepancies for our key nodes all varied by less than 10%, except for the crown ages of Xylocopini and Manueliini (Table 1). For these two latter tribes, the penalized likelihood analysis gave crown ages that were approximately 10 My older than the BEAST analyses.

Maximum likelihood (ML) and Bayesian MCMC analyses based on 41 extant Xylocopinae species for which social data are available (Table S1) and using 2000 post-burnin chronograms indicated that social nesting was the ancestral trait for the Xylocopinae, with an estimated mean probability of P = 0.997 from ML analyses and P = 0.821 from the MCMC analyses (both averaged over the 2000 chronograms). The MCMC probabilities for this node as well as the nodes for each of the Xylocopinae tribes are summarized as pie charts in Figure 1. A Bayes Factor test, where social and solitary nesting were separately constrained as ancestral conditions, favoured social nesting as the ancestral condition (BF = 4.65).

We used maximum likelihood analyses to infer maximum colony size in the ancestral Xylocopinae lineage, along with λ, κ and δ values [35]. λ is a measure of how well phylogeny explains variation in a trait, with values close to 1 indicating a strong phylogenetic signal. κ provides a measure of how changes in a trait vary with branch lengths, and δ provides a measure of whether rates of change in a trait vary with distance from the root [35]. Our analyses indicated a strong effect of phylogeny on colony size (mean λ = 0.99) and an ancestral maximum size of 3 females per nest (Table S1). Interestingly, the mean κ value was 2.29, indicating relatively greater change with increasing branch lengths, rather than a model of punctuated evolution, and the mean δ was 0.88, suggesting gradualism without a tendency for accelerated change either close to the root or close to the present.

Lastly, our analyses indicated an extremely low probability for castes being present in the Xylocopinae root node (P = 0.023). There was moderately low probability for true castes being present in the root nodes of the two genera containing species with true castes, *Exoneurella* (P = 0.211) and *Hasinamelissa* (P = 0.303), and extremely low probability for true castes being present in the most recent common ancestor (MRCA) of these two clades (P = 0.021). Combined with our chronogram analyses, this indicates two origins of true castes, both occurring more recently than the estimated age of 44.3 Mya for the MRCA of *Hasinamelissa* and *Exoneurella*.

## Discussion

The bee tribe Manueliini contains only three species, and the two species whose nesting biology is well known are both strictly solitary. A basal position of Manueliini in the Xylocopinae would make it more likely that solitary nesting is the ancestral trait for this subfamily. However, our analyses overwhelmingly indicate that Xylocopini comprises the basal tribe within the Xylocopinae, supporting a recent study of the Apidae using five gene fragments from a wide sample of apid tribes [7], but contrasting with some earlier studies that employed much narrower taxon representation and smaller character sets (e.g. [25], [28]). Our phylogenetic results are therefore important for inferring social evolution in the Xylocopinae because we can now be certain that the tribe Manueliini is not basal.

Our maximum likelihood analyses indicated an extremely high probability (P = 0.997) that social living is ancestral for the Xylocopinae. Our MCMC analyses indicated a lower, but still substantial, probability (P = 0.821) for sociality being ancestral, and a Bayes Factor test indicated this was positive, verging on strong, support. We note that that these probabilities are predicated on an absence of social/solitary nesting for many species in our analyses, particularly in the tribes Xylocopini and Ceratinini. However, there are reasons to believe that many of the species for which we have missing data may be social. For example, in a review that covered evidence for solitary or cooperative nesting in *Xylocopa*, Vicidomini [30] listed 13 social nesters from nine subgenera, and 12 solitary nesters also from nine subgenera, but two of these subgenera (*Koptortosoma* and *Neoxylocopa*) contained both solitary and social species. In addition to species covered in Vicidomini's study, social nesting has been reported in another four *Xylocopa* species, *X.* (*Koptortosoma*) *pubescens* [36], *X.* (*Ctenoxylocopa*) *sulcatipes* [37], *X.* (*Lestis*) *bombylans* and *X.* (*L.*) *aeratus* [38]. Likewise, *Ceratina* species have long been considered solitary with few studies reporting social traits [13]–[15], [39]. However, recent studies have shown sociality in *C. (Pithitis) smaragdula, C. (Ceratinidia) nigrolateralis, C. (C.) accusator* and *C. (Neoceratina) australensis* [40]–[44]. When combined, multiple studies therefore indicate that social nesting in *Xylocopa* and *Ceratina* is both frequent and taxonomically widespread.

Given the above considerations, our results have important implications for understanding social evolution in bees. Cardinal and Danforth [7] showed that eusociality evolved once in the corbiculate bees, but it is not known if this origin was preceded by a period of less complex sociality in an ancestral lineage and, if so, what kind of social structure that ancestor may have had. However, the Xylocopinae, which is nested within the same subfamily Apinae as the corbiculates, provides numerous examples of simple forms of sociality as well as some socially complex species [22]. Our analyses show that a simple form of sociality is ancestral for the Xylocopinae and that sociality has been in place for about 100 My. A number of important consequences flow from this conclusion, which we now discuss.

Eusociality in the corbiculates evolved some 87 Mya, but that was only 10 My after divergence of that group from its sister clade, the solitary-nesting tribe Centridini [7]. This means that eusociality in the corbiculates would have been preceded by, at most, 10 My of evolution involving simple forms of sociality. In contrast, for the Xylocopinae the lag time from simple ancestral sociality (about 100 Mya) to eusociality involving true worker castes (less than the most recent common ancestor of *Exoneurella* and *Hasinamelissa*, ca. 45 Mya) was about 55 My and potentially much longer, depending on when worker castes evolved in the lineages leading to *E. tridentata* and *H. minuta*. Combined, these findings indicate that complex eusociality can evolve from a non-social ancestor comparatively quickly, as in the corbiculates, or very slowly despite a very long period of simple sociality, as in the Xylocopinae. This disparity in lag times from solitary to eusocial suggests that origins of eusociality cannot be simply explained by the length of time that evolution can operate on primitively social precursors.

Attempts to understand how eusociality has evolved, or why it has evolved so few times, have often involved identifying so-called pre-adaptations or conditions for eusociality (e.g. [3]–[5]). These all involve features that are present in social species of Xylocopinae, such as overlap of generations, use of a defensible nest with resources concentrated in that nest, opportunities for kin to cooperate in nest use, and opportunities for such cooperation to enhance nest defence or resource acquisition. For example, cooperative nesting leads to almost ubiquitous decreases in rates of brood loss in allodapines [32]. Increased defence of brood is also widely reported for social species of *Xylocopa* and *Ceratina* [13], [25], [40], [42], [45]–[47]. Overlap of generations has also been reported in all social species in the Xylocopinae studied to date [17]–[19], [32], [48], [49], and the only studies of Xylocopinae to report cooperative nesting among unrelated females involve artificially forced associations in *Ceratina* (e.g. [15], [25], [43], [50]). Consequently, these conditions for eusociality, and the length of time that simple sociality has been in place, are not sufficient to explain why eusociality has evolved so infrequently in the Xylocopinae. It therefore seems likely that much more stringent conditions are operating.

Schwarz et al. [51] argued that, among allodapines, the evolution of sterile workers in *Exoneurella tridentata* and *Hasinamelissa minuta* is linked to harsh environmental conditions that simultaneously limit opportunities for dispersal and opportunities for subordinate females to survive long enough to assume a position of reproductive dominance. This argument falls broadly into an approach that posits ‘causal mosaics’ (*sensu* Crespi [5]) – specific combinations of selective factors and life history traits that may be able to explain individual origins of eusociality where more general hypotheses have little or no predictive value. Dew et al. [52] have shown that within the allodapines, large colony size in *E. tridentata* represents a threshold event rather than the result of gradual evolutionary change within that tribe. This is also concordant with the idea that some rare coincidence of selective factors is required for eusociality to evolve, rather than eusociality representing an outcome from a gradual and long-term evolutionary trend.

The notion that the evolution of true worker castes requires a highly unusual mosaic of selective and life history conditions could potentially explain why its origins have been so few and yet so widely distributed over time. If eusociality requires a gradual accumulation of more and more complex traits we might expect to see origins becoming more frequent closer to the present, but this does not seem to be the case. If causal mosaics have a different composition of facilitating factors for each inferred origin, they will not permit straightforward statistical assessment, and recent molecular studies on at least bees [7], [53], indicate that the number of eusocial origins now known is much smaller than earlier studies suggested [2], [17], [39], [54].

Whilst recent molecular phylogenetic studies have decreased the number of eusocial origins available for comparative studies, they have increased the number of known reversions to solitary living. There are 12 inferred losses of sociality in halictines [53], one loss in the corbiculate bees [7], and our study indicates at least four losses in the Xylocopinae. Because the large majority of Xylocopini and Ceratinini species have received no detailed studies of nesting biology, it is likely that there are more than four losses of sociality in the Xylocopinae. In the Xylocopini reversals to solitary nesting (as in *X. violacea* [30] and *X. caffra* [55]) occur in species that are found in more temperate climates and coincide with transitions from multivoltine to univoltine, indicating that climate may play a role here. Likewise, reversals to solitary nesting in *Ceratina (Zadontomerus)* occur in species that do not reuse nesting substrates [20], [49]. Life history traits such as obligate nest dispersal and univoltine colony cycles limit opportunities for overlapping generations and cooperative brood care. Paradoxically, comparative studies focussed on losses of sociality may be our best strategy for understanding the origin of sociality.

## Methods

### Taxon sampling

Taxa and sampling localities along with NCBI accession numbers are listed in Table S1. All new data have been deposited in Genbank accession numbers JQ230006–JQ230057. Our ingroup comprised 70 species sampled from all four tribes of the Xylocopinae [56]: Allodapini (22 species), Ceratinini (31 species), Manueliini (3 species), and Xylocopini (11 species). Our taxa covered all species of Manueliini, and choice of species in the three other tribes was based on availability of sequence data and the desirability of representing as wide a range as possible of the major intra-tribal clades identified by previous studies [31]–[33].

### DNA sequences

Four gene fragments were used for phylogenetic analyses: two mitochondrial genes cytochrome oxidase subunit 1 (COI - 1279 base pairs) and cytochrome b (cytb - 428 base pairs), and two nuclear genes,the F1 and F2 copies of Elongation Factor-1α (EF-1α), with 460 and 772 base pairs respectively. DNA extraction, PCR amplification and sequencing were performed as described in Leys et al. [31], Schwarz et al. [32] and Rehan et al. [33]. Most sequences were from previous studies and references for their sources, along with accession numbers for newly sequenced species, are listed in the supplementary material (Table S1). The intron region of the F2 copy of EF-1α was largely unalignable and was not included in the analyses.

### Phylogeny construction

We used three methods to explore tribal relationships in the Xylocopinae. Firstly we used a Bayesian Monte Carlo Markov Chain (MCMC) approach, implemented in BEAST 1.6.2 [57] with a relaxed log-normal clock model. For this analysis we separately combined the two mitochondrial genes and the two nuclear genes and then each group was partitioned into 1^st^ plus 2^nd^, and 3^rd^ codon positions, producing a total of six partitions. A GTR+I+Γ model was fitted to each partition because this is the most general model available and effectively allows more restrictive models when some model parameters converge to similar values. For tree construction we used a Yule process with the prior for birthrate drawn from a uniform distribution bounded by 0 and infinity. We used a total of 20 million generations, sampling every 1000^th^ generation and with a burnin of 10 million. Stationarity in models was assessed by plotting parameter values in the program Tracer 1.4.1 [57].

As a check that the phylogeny produced from our BEAST analysis was robust to different analytical approaches, we also carried out Bayesian analyses in MrBayes version 3.1.2 [58], BayesPhylogenies 1.1 [59], and a maximum parsimony analysis in PAUP* v4.0b10 [60].

For MrBayes analyses we used the same gene partitioning scheme as for our BEAST analyses, leading to six partitions . We used default MrBayes priors, with a GTR+I+Γ model for each partition, and partitions were unlinked for all substitution model parameters. Two analyses were run in parallel, each for 20 million generations with 16 chains, sampling every 1000^th^ generation. Stationarity in model parameters was assessed by examining the average standard deviation of split frequencies (ASDSF), along with trace plots of log likelihood (LnL) values along with other parameters, such as transition rates and base composition frequencies for each partition, again using Tracer 1.4.1 [57]. We chose a burnin of 15 million, well after stationarity was reached, so that the consensus phylogram and posterior probabilities were based on 10,000 post-burnin trees.

BayesPhylogenies [59] implements an MCMC method allowing a mixture model where multiple models of sequence evolution can be applied to nucleotides without having to partition data *a priori*. In our analyses, this served to check whether tribal relationships inferred from the BEAST and MrBayes analyses were dependent on the partitions that we set prior to analysis. For our BayesPhylogenies analysis we chose to use four patterns of sequence evolution, each with a separately estimated GTR model, with gamma rate heterogeneity and base frequencies estimated separately for each of the four models. We used three chains run for five million iterations sampling every 500^th^ generation. Stationarity in the models was assessed using Tracer to examine parameters across sampled generations, as was done for the MrBayes analysis, and we used a burnin of 4 million iterations.

Our maximum parsimony (MP) analysis was implemented in PAUP*[60], as a further check for robustness of tribal relationships. MP analysis employed 50 random sequence addition heuristic searches, holding 10 trees at each step. Node support was assessed using bootstrap analysis with the same heuristic search procedure, with 1000 bootstrap pseudoreplicates.

### Estimating Divergence Ages

Chronograms in BEAST were produced using a log normal relaxed clock model and default priors. For the MrBayes analysis, we produced chronograms using Sanderson's penalised likelihood (PL) transformation of phylograms, enabled in the program r8s [61], and this was applied to both the consensus phylogram from our MrBayes analysis, as well as 300 random postburnin phylograms from the same analysis.

The only reliable internal calibration point available for the Xylocopinae is the presence of Boreallodapini (extinct sister clade to Allodapini with Ceratinini representing the next-most basal divergence) fossils from Baltic amber dated at 45.1 Mya [20]. In initial analyses we therefore explored the effect of setting a minimum divergence age of 45 Mya between Ceratinini and Allodapini and using a variety of root node ages (uniting the corbiculates with the Xylocopinae) ranging from 90 Mya to 120 Mya. Our chronograms from both the BEAST and MrBayes+r8s analyses were subsequently calibrated by setting a fixed date for the root node and we varied this from 90 Mya to 120 Mya and also included a set date of 107 Mya corresponding to the date estimated by Cardinal and Danforth [7].

### Social evolution analysis

Each species was coded as either social, solitary or as unknown based on a review of the current literature (ESM Table 1). We use the term ‘social’ here in a broad sense to include species where two or more adult females are present in a nest while eggs are being laid and brood are being actively provisioned. Our use of the term sociality therefore covers all forms of sociality that have previously been designated as eusocial, semisocial and quasisocial [2], [39], [62] but does not include subsocial colonies with only a single adult female, or communal colonies. We do not use the term ‘sociality’ here to imply that all such nestmates are actively involved in rearing brood, but rather that reproductive females tolerate the presence of other adult females whether the latter help in brood rearing or not. Such cases comprise a clear ‘pre-adaptation’ to sociality involving worker-like behaviour, since selection is able to operate on already-present associations to produce a division of labour. We coded the two *Inquilina* social parasites as social, rather than solitary, because this genus is derived from a social ancestor (its host clade) and because their mode of living entails important social traits, including nestmate recognition, integrating with the host social hierarchy, soliciting trophallaxis via social communication, and having their brood reared by alloparental care rather than kleptoparastism [63]. We coded the social status of the rare Middle Eastern allodapine *Exoneuridia hakkariensis* as missing because the nesting biology of this species has never been described.

We used both maximum likelihood (ML) and MCMC methods, both implemented in BayesTraits [35], [64] to infer the ancestral states (solitary or social) for each tribe and for the root node of the Xylocopinae. Species for which we had sequence data but not social data were included in phylogenetic trees but their social states were treated as missing. To account for the effects of phylogenetic uncertainty, analyses were applied to both the consensus phylogram from the BEAST analysis, as well as 2000 post-burnin chronograms.

For ML analyses we calculated the probability of any one state being ancestral for the Xylocopinae root node as well as the root nodes for each of the four tribes. Support for any one state can be gauged using a likelihood ratio test (LRT) where the LR = 2(LnL(better fitting model)−LnL(worse fitting model)) [35]. There is no natural way to combine ML ancestral state analyses from multiple chronograms into a single assessment [60], so we examined results from 2000 post-burnin chronograms as well as the average of these results.

For MCMC analyses, multiple post-burnin chronograms can be combined in a single analysis so that phylogenetic uncertainty is taken into account when estimating posterior probabilities over the tree samples [35], and for this we used the same 2000 post-burnin chronograms as for the ML analysis. Priors for character state transition rates were based on the distribution of rates from the ML analyses, which suggested zero-truncated exponential distributions, and these were seeded using a reverse jump hyperprior. For each analysis we explored a range of rate deviation values with the criterion that acceptance rates varied between 0.2–0.4. Harmonic means of the LnL were examined for multiple runs to determine an appropriate burnin and total number of iterations. Following these multiple runs, we used a burnin of 100 million iterations and a total run of 1 billion generations, sampling every 100,000^th^ iteration. Finally we statistically assessed the likelihood of the root node being social or non-social by fixing (‘fossilising’) this node for each state and then comparing the model likelihoods using a Bayes Factor test, where the BF = 2(LnL(best fitting model)−LnL(worse fitting model)), where LnL is calculated as the harmonic mean of post-burnin log likelihoods, and values of BF>2 indicate support for the better fitting model, and values >5 indicate strong support (e.g. [64]).

We also examined the evolution of maximum colony size (maximum number of adult females per nest, excluding callows, in nests where brood were being actively reared) and the presence of morphologically-based castes. Maximum colony sizes for included species are given in table S1 along with references for data sources. Morphologically-based castes have been reported for only two species in the Xylocopinae, *Hasinamelissa minuta* and *Exoneuridia tridentata* [51], so all other species in our data set where nesting biology has been described were coded as lacking such castes. Ancestral colony size was inferred using the MCMC option in the Continuous module of BayesTraits [28]. We used a burnin of 10 million generations and a total of 1 billion iterations, sampling every 1 millionth iteration to reduce autocorrelation of sampled values. Stationarity in the model was assessed by plotting the harmonic mean of the LnL, and the ancestral value was based on the mean of sampled values after stationarity. The presence of morphology-based castes at the root and internal nodes was assessed using the BayesMultiState module in BayesTraits. We used the same burnin and total iterations as for our Continuous analysis, and a rate deviation of 60 to ensure acceptance rates of between 0.2 and 0.4, as recommended [28]. Probabilities for the presence/absence of castes at chosen nodes was estimated as means for sampled iterations after model stationarity, as determined from plots of the harmonic mean LnL. Both the Continuous and MultiState analyses were carried out three times to check for consistency in outcomes.

## Supporting Information

Figure S1 **Chronogram obtained from a penalised likelihood transformation of the consensus phylogram obtained from a MrBayes analysis.**
(DOC)

Figure S2 **Consensus phylogeny obtained from the BayesPhylogenies analysis, along with posterior probabilities for all nodes.**
(DOCX)

Figure S3 Bootstrap consensus tree from a maximum parsimony analysis implemented in PAUP*.
(DOCX)

Table S1 **Genbank accession numbers and social status and references for this status for Xylocopinae species in our study.**
(DOC)
