# Supplementary material for: A Mid-Cretaceous Origin of Sociality in Xylocopine Bees with Only Two Origins of True Worker Castes Indicates Severe Barriers to Eusociality
Source: PLoS One. 2012 Apr 12;7(4):e34690. doi: 10.1371/journal.pone.0034690 (PMC3325255; doi:10.1371/journal.pone.0034690)

**Figure S1:** Chronogram obtained from a penalised likelihood transformation of the consensus phylogram obtained from a MrBayes analysis. Posterior probability support for each node is indicated by numbers next to nodes for nodes where PP support was <98%. Social species are coloured red, solitary species are blue and species where social status are unknown are black. The two species with true worker castes are indicated by black rectangles. The relative probabilities of social and solitary as states for key internal nodes are represented by pie charts, where probabilities are means of maximum likelihood values based on 300 postburnin chronograms. For Xylocopini and Ceratinini, which are both monogeneric, we have used the subgenus rather than genus name in binomens.


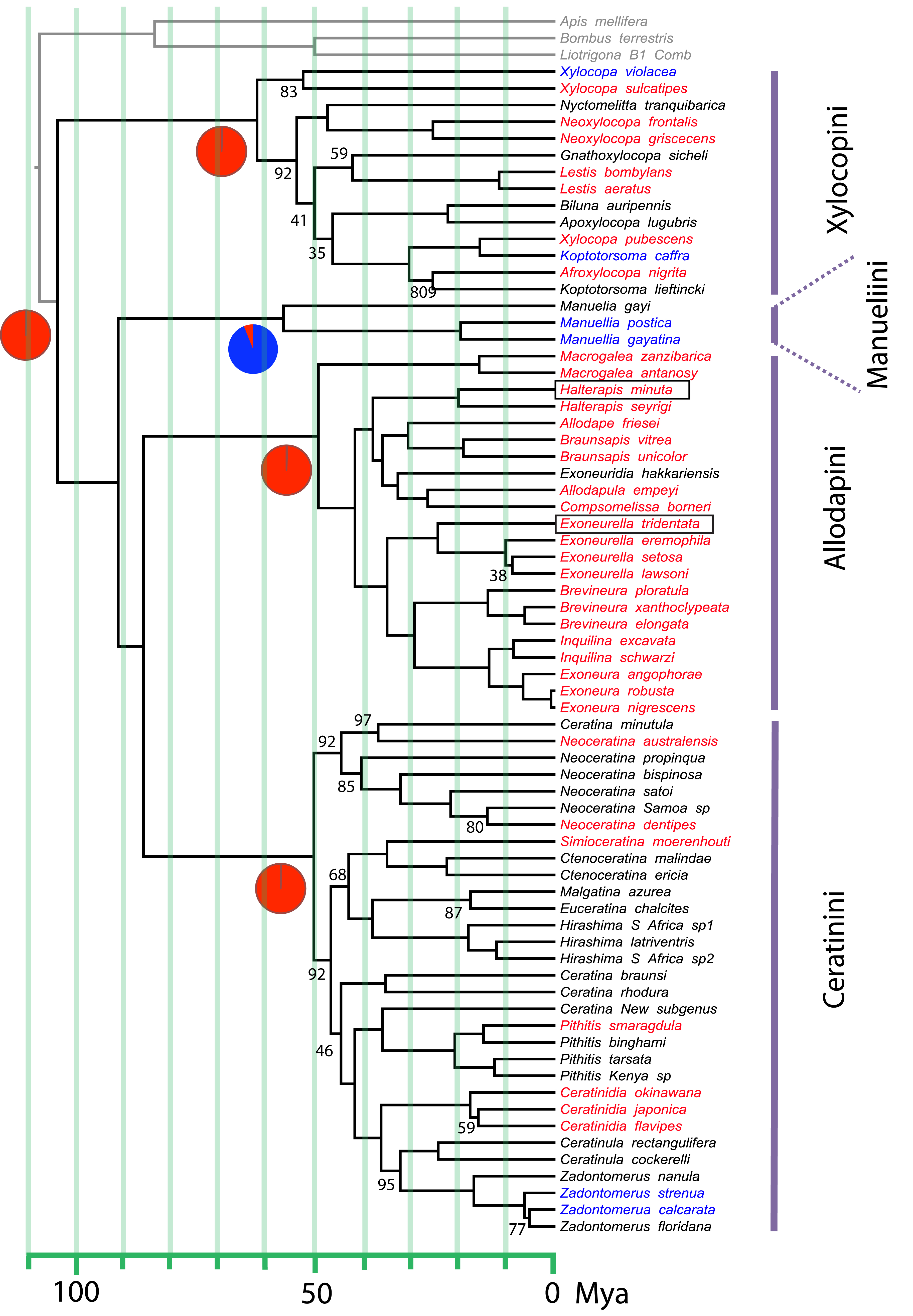

Supplement: Figure S1 — Chronogram obtained from a penalised likelihood transformation of the consensus phylogram obtained from a MrBayes analysis. (DOC) [file pone.0034690.s001.doc]
