# Supplementary material for: A Mid-Cretaceous Origin of Sociality in Xylocopine Bees with Only Two Origins of True Worker Castes Indicates Severe Barriers to Eusociality
Source: PLoS One. 2012 Apr 12;7(4):e34690. doi: 10.1371/journal.pone.0034690 (PMC3325255; doi:10.1371/journal.pone.0034690)

**Figure S2:** Consensus phylogeny obtained from the BayesPhylogenies analysis, along with posterior probabilities for all nodes. Outgroup is indicated by grey branches.


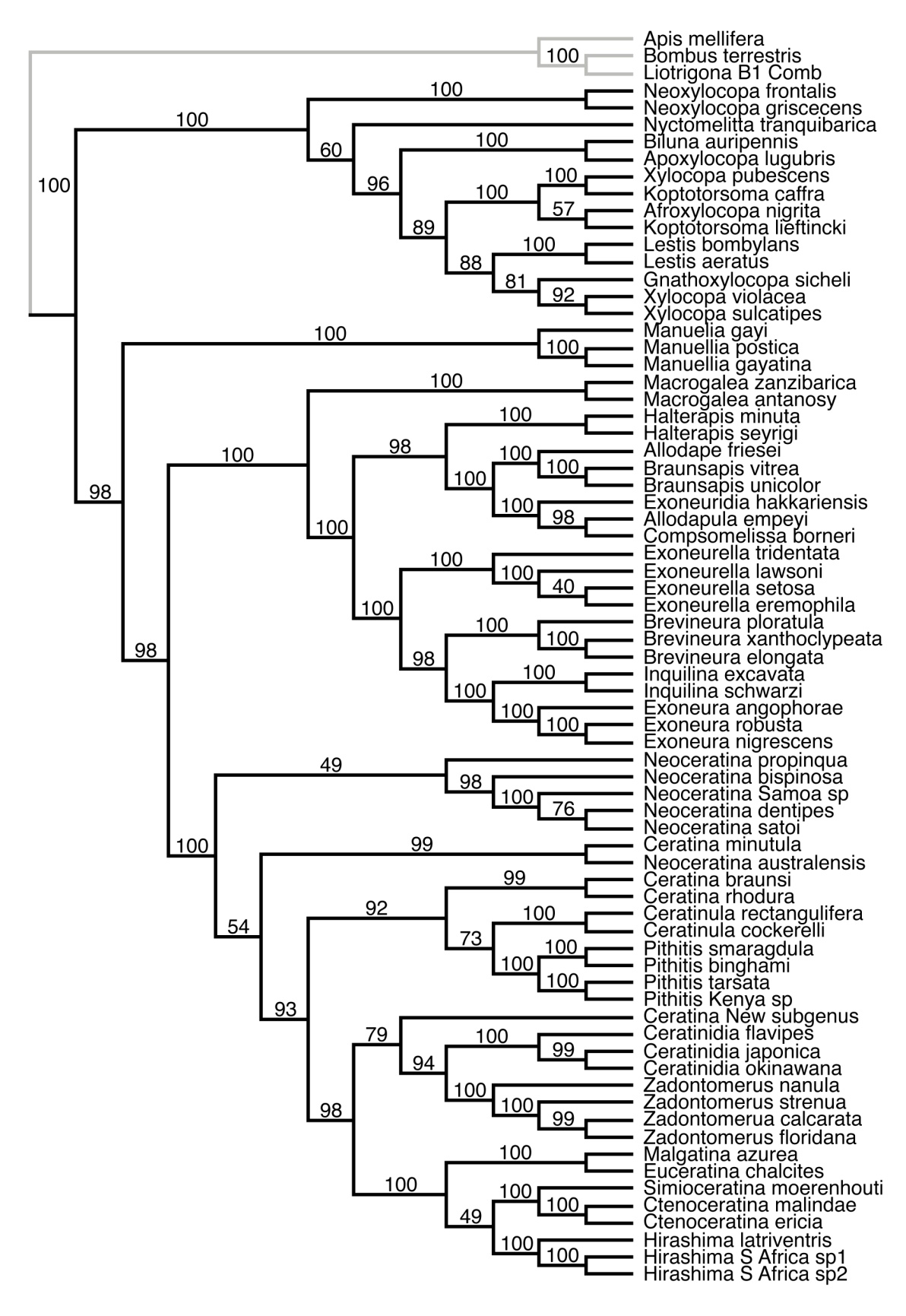

Supplement: Figure S2 — Consensus phylogeny obtained from the BayesPhylogenies analysis, along with posterior probabilities for all nodes. (DOCX) [file pone.0034690.s002.docx]
