# Supplementary figures and images for: A Mid-Cretaceous Origin of Sociality in Xylocopine Bees with Only Two Origins of True Worker Castes Indicates Severe Barriers to Eusociality
Source: PLoS One. 2012 Apr 12;7(4):e34690. doi: 10.1371/journal.pone.0034690 (PMC3325255; doi:10.1371/journal.pone.0034690)

**Figure S3:**  Bootstrap consensus tree from a maximum parsimony analysis implemented in PAUP*.


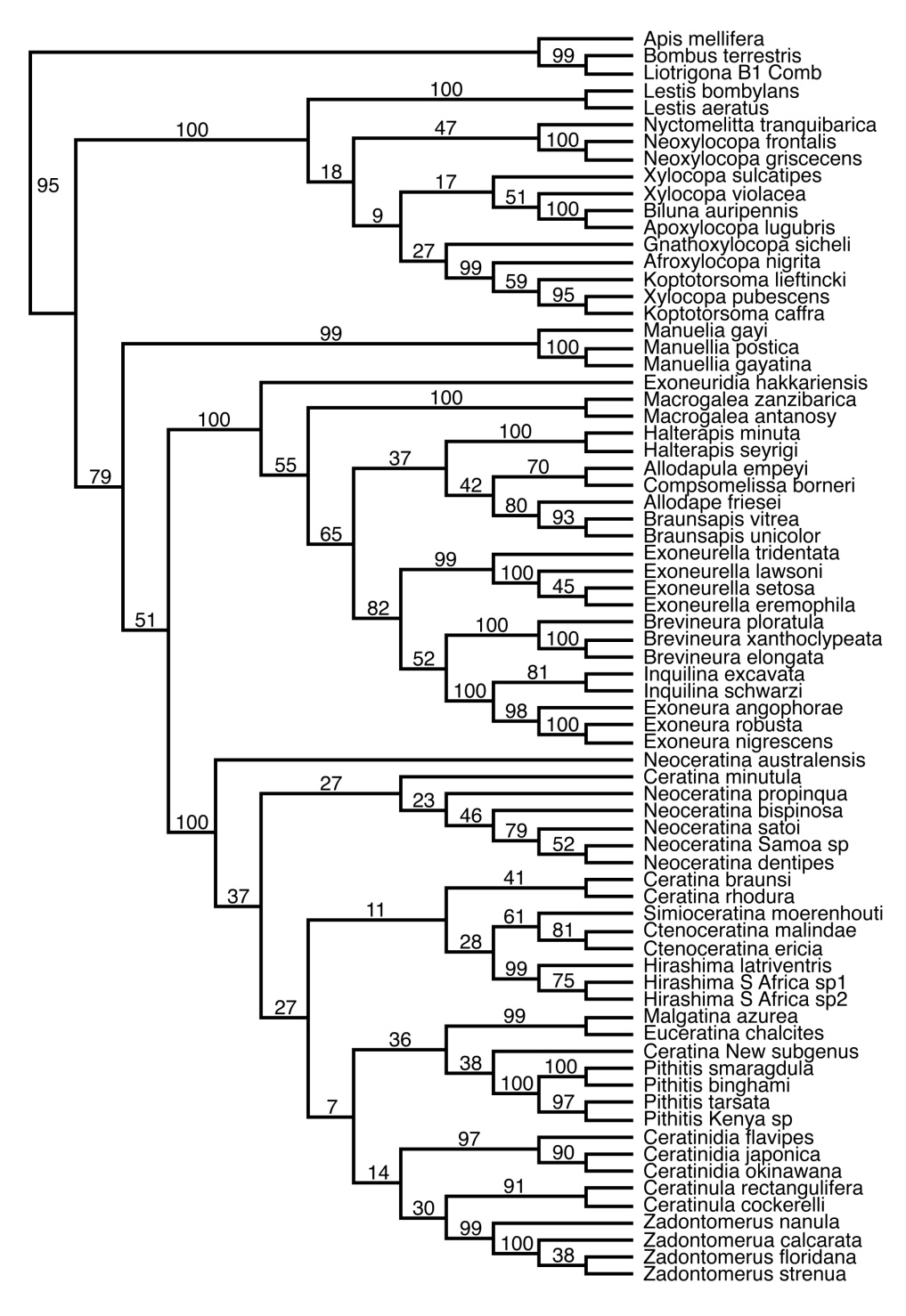

Supplement: Figure S3 — Bootstrap consensus tree from a maximum parsimony analysis implemented in PAUP*. (DOCX) [file pone.0034690.s003.docx]
