# Supplementary material for: A Mid-Cretaceous Origin of Sociality in Xylocopine Bees with Only Two Origins of True Worker Castes Indicates Severe Barriers to Eusociality
Source: PLoS One. 2012 Apr 12;7(4):e34690. doi: 10.1371/journal.pone.0034690 (PMC3325255; doi:10.1371/journal.pone.0034690)
Supplement: Table S1 — Genbank accession numbers and social status and references for this status for Xylocopinae species in our study. (DOC) [file pone.0034690.s004.doc]

**Table S1:** Genbank accession numbers and social status and references for this status for Xylocopinae species in our study. Dashes (-) indicate no data. For species included in analyses of social data, social status and maximum colony are also given.

| **Tribe** | **Species** | **COI** | **Cytb** | **EF-1α (F1)** | **EF-1α (F2)** | **Social/**  **solitary** | **Max colony size** | **References for social data** |
| --- | --- | --- | --- | --- | --- | --- | --- | --- |
| Allodapini | *Macrogalea zanzibarica* | AJ416811.1 / DQ149679.1 | AY625455.1 | DQ149699.1 | DQ149723.1 | Soc | 10 | Tierney et al. 2002 |
|  | *Macrogalea antanosy* | DQ149680.1 | DQ149612.1 | - | DQ149724.1 | Soc | 6 | Smith and Schwarz 2006 |
|  | *Exoneurella tridentata* | AJ416793.1 / DQ149665.1 | AF072670.1 | AJ416766.1 | DQ149710.1 | Soc | 37 | Hurst 2001 |
|  | *Exoneurella setosa* | AJ416795.2 / DQ149666.1 | AF072671.1 | AJ416768.1 | DQ149711.1 | Soc | 7 | Neville et al. 1998 |
|  | *Exoneurella lawsoni* | AF072661.1 / DQ149668.1 | AF02668.1 | AJ416765.1 | DQ149713.1 | Soc | 5 | Michener 1964 |
|  | *Exoneurella eremophila* | AF072662.1 / DQ149667.1 | AF072669.1 | DQ149696.1 | DQ149712.1 | Soc | 5 | Hogendoorn et al. 2001 |
|  | *Brevineura xanthoclypeata* | AJ416798.1 / DQ149672.1 | AJ416826.1 | AJ416771.1 | DQ149717.1 | Soc | 7 | Tierney et al. 1997 |
|  | *Brevineura elongata* | AJ416799.1 / DQ149673.1 | AJ416827.1 | AJ416772.1 | DQ149718.1 | Soc | 8 | Joyce and Schwarz 2006 |
|  | *Exoneura angophorae* | AJ416786.1 / DQ149660.1 | AJ416814.1 | AJ416759.1 | DQ149705.1 | Soc | 5 | Schwarz et al. 1996 |
|  | *Compsomelissa borneri* | AJ416812.1 | AJ416840.1 | AJ416784.1 | DQ149719.1 | Soc | 3 | Michener 1971 |
|  | *Exoneura robusta* | AJ416787.1 / DQ149661.1 | AJ416815.1 | EJ416760.1 | DQ149706.1 | Soc | 7 | Schwarz 1986 |
|  | *Exoneura nigrescens* | AJ416789.1 / DQ149662.1 | AJ416817.1 | AJ416762.1 | DQ149707.1 | Soc | 7 | Bull and Schwarz 1997 |
|  | *Exoneuridia hakkariensis* | DQ149678.1 | DQ149691.1 | DQ149698.1 | DQ149722.1 | - | - |  |
|  | *Hasinamelissa minuta* | DQ149676.1 | DQ149689.1 | - | DQ149720.1 | Soc | 6 | Schwarz et al. 2005 |
|  | *Hasinamelissa seyrigi* | DQ149677.1 | DQ149690.1 | - | DQ149721.1 | Soc | 7 | Chenoweth and Schwarz 2007 |
|  | *Allodapula empeyi* | DQ149659.1 | DQ149684.1 | DQ149695.1 | DQ149704.1 | Soc | 3 | Dew et al. 2011 |
|  | *Allodape friesei* | DQ149656.1 | DQ149683.1 | DQ149694.1 | DQ149701.1 | Soc | 3 | Michener 1971; Schwarz & Tierney, unpub. obs |
|  | *Braunsapis vitrea* | AJ416808.1 / DQ149657.1 | AJ416836.1 | AJ416781.1 | DQ149702.1 | Soc | 3 | Aenmey et al. 2006 |
|  | *Braunsapis unicolor* | AF072659.1 / DQ149658.1 | AF072666.1 | AJ416776.1 | DQ149703.1 | Soc | 7 | Dew et al. 2011 |
|  | *Inquilina excavate* | DQ149669.1 | DQ149687.1 | DQ149697.1 | DQ149714.1 | Soc (Para.) | - | Michener 1965 |
|  | *Inquilina schwarzi* | DQ149670.1 | U56094.1 | AJ416764.1 | DQ149715.1 | Soc (Para.) | - | Smith and Schwarz 2006 |
| Ceratinini | *Ceratina (Neoceratina) minutula* | GU321671 | GU321601 | - | GU321643 | - | - | - |
|  | *C. (Neoceratina) australensis* | GU321616 | GU321553 | JQ230015 | GU321616 | Soc | 2 | Rehan et al. 2010 |
|  | *C. (Neoceratina) propinqua* | GU321520 | GU321581 | JQ230017 | GU321655 | - | - | - |
|  | *C. (Neoceratina) bispinosa* | GU321521 | GU321587 | JQ230018 | GU321657 | - | - | - |
|  | *C. (Neoceratina) satoi* | GU321518 | GU321583 | JQ230019 | GU321653 | - | - |  |
|  | *C. (Neoceratina)* Samoa sp | JQ230010 | - | JQ230014 | JQ230055 | - | - | - |
|  | *C. (Neoceratina) dentipes* | GU321516 | GU321581 | JQ230016 | GU321651 | Soc | 2 | Rehan et al. 2009 |
|  | *C. (Ceratina) braunsi* | GU321532 | GU321597 | JQ230039 | - | - | - | - |
|  | *C. (Ceratina) rhodura* | GU321537 | GU321602 | JQ230040 | GU321672 | - | - | - |
|  | *C. (Simioceratina) moerenhouti* | GU321486 | GU321557 | JQ230025 | GU321621 | Soc | 2 | Daly et al. 1988 |
|  | *C. (Ctenoceratina) malindae* | GU321496 | GU321564 | JQ230026 | GU321631 | - | - | - |
|  | *C. (Ctenoceratina) ericia* | GU321489 | GU321559 | JQ230027 | GU321624 | - | - | - |
|  | *C. (Malgatina) azurea* | GU321481 | GU321553 | JQ230023 | GU321616 | - | - | - |
|  | *C. (Euceratina) chalcites* | GU321485 | - | JQ230024 | GU321620 | - | - | - |
|  | *C. (Hirashima)* S Africa sp1 | GU321483 | GU321555 | JQ230033 | GU321618 | - | - | - |
|  | *C. (Hirashima)* S Africa sp2 | GU321511 | GU321576 | JQ230034 | GU321646 | - | - | - |
|  | *C. (Hirashima) lativentris* | GU321514 | GU321579 | JQ230032 | GU321649 | - | - | - |
|  | *C. (*New Subgenus) | - | GU321586 | JQ230041 | GU321656 | - | - | - |
|  | *C. (Pithitis) smaragdula* | GU321523 | GU321589 | JQ230035 | GU321659 | Soc | 2 | Rehan et al. 2009 |
|  | *C. (Pithitis) binghami* | GU321526 | GU321592 | JQ230037 | GU321662 | - | - | - |
|  | *C. (Pithitis) tarsata* | GU321530 | GU321596 | JQ230036 | GU321666 | - | - | - |
|  | *C. (Pithitis)* Kenya sp | GU321527 | GU321593 | JQ230038 | GU321663 | - | - | - |
|  | *C. (Ceratinidia) okinawana* | GU321478 | GU321550 | JQ230002 | GU321613 | Soc | 2 | Sakagami & Maeta 1989 |
|  | *C. (Ceratinidia) japonica* | GU321470 | GU321542 | JQ230020 | GU321605 | Soc | 2 | Sakagami and Maeta 1984 |
|  | *C. (Ceratinidia) flavipes* | JQ230011 | JQ230006 | JQ230022 | - | Soc | 2 | Sakagami and Maeta 1977, 1987 |
|  | *C. (Ceratinula) rectangulifera* | JQ230013 | JQ230008 | - | JQ230057 | - | - | - |
|  | *C. (Ceratinula) cockerelli* | GU321641 | - | - | GU321641 | - | - | - |
|  | *C. (Zadontomerus) nanula* | JQ230012 | JQ230007 | JQ230031 | JQ230056 | - | - | - |
|  | *C. (Zadontomerus) strenua* | GU321505 | GU321572 | JQ230030 | GU321640 | Sol | 1 | Kislow 1976 |
|  | *C. (Zadontomerus) calcarata* | GU321499 | GU321567 | JQ230028 | GU321634 | Sol | 1 | Rehan & Richards 2010 |
|  | *C. (Zadontomerus) floridana* | GU321487 | GU321558 | JQ230029 | GU321622 | - | - | - |
| Manueliini | *Manuelia gayi* | HM461878 | HM461881 | HM461884 | - | - | - | - |
|  | *Manuelia gayatina* | HM461879 | HM461882 | HM461885 | GU321604 | Sol | 1 | Daly et al 1987 |
|  | *Manuelia postica* | HM461880 | HM461883 | HM461886 | - | Sol | 1 | Flores Prado et al. 2008 |
| Xylocopini | *Xylocopa (Nyctomelitta) tranquebarica* | AY005224.1 | AY005251.1 | AY005278.1 | JQ230044 | - | - | - |
|  | *X. (Neoxylocopa) frontalis* | AY005248.1 | AY005275.1 | AY005302.1 | JQ230049 | Soc | 2 | Camilo & Garofalo 1982, 1989 |
|  | *X. (Neoxylocopa) grisescens* | JQ230009 | - | - | JQ230054 | Soc | 2 | Camilo & Garofalo 1982, 1989 |
|  | *X. (Biluna) auripennis* | AY005225.1 | AY005252.1 | AY005279.1 | JQ230046 | - | - | - |
|  | *X. (Apoxylocopa) lugubris* | EU180090.1 | EU180106.1 | EU180118.1 | JQ230047 | - | - | - |
|  | *X. (Afroxylocopa) nigrita* | AY005238.1 | AY005265.1 | AY005292.1 | JQ230045 | Soc | 2 | Anzenberger 1977 |
|  | *X. (Koptortosoma) caffra* | EU180083.1 | EU180102.1 | EU180112.1 | JQ230050 | Sol | 2 | Watmough 1983 |
|  | *X. (Koptortosoma) lieftincki* | AY005235.1 | AY005262.1 | AY005289.1 | JQ230051 | - | - | - |
|  | *X. (Gnathoxylocopa) sicheli* | AY005230.1 | AY005257.1 | AY005284.1 | JQ230048 | - | - | - |
|  | *X. (Lestis) bombylans* | AY005227.1 | AY005254.1 | AY005281.1 | JQ230052 | Soc | 3 | Steen 2000 |
|  | *X. (Lestis) aeratus* | EU180091.1 | EU180107.1 | EU180119.1 | JQ230053 | Soc | 3 | Steen 2000 |
|  | *X.(Xylocopa) violacea* | AY005226.1 | AY005253.1 | AY005280.1 | JQ230042 | Sol | 1 | Vicidomini 1996 |
|  | *X.(Ctenoxylocopa) sulcatipes* | AY005233.1 | AY005260.1 | AY005287.1 | - | Soc | 2 | Gerling et al. 1983 |
|  | *X.(Koptortosoma) pubescens* | AY005236.1 | AY005263.1 | AY005290.1 | JQ230043 | Soc | 2 | Gerling et al. 1981 |

**Supplementary References**

Aenmey, TK, Tierney SM, Pillay N, Schwarz MP 2006 Nesting biology of an African allodapine bees *Braunsapis vitrea*: female biased sex allocation in the absence of worker-like behavioural castes *Ethol Ecol Evol* **18**, 205-220

Anzenberger G 1977 Ethological study of African carpenter bees of the genus *Xylocopa* (Hymenoptera, Antophoridae) *Z Tierpsychol* **44**, 337-374

Bull NJ, Schwarz MP 1997 Rearing of non-descendant offspring in an allodapine bee, *Exoneura bicolor* Smith (Hymenoptera: Apidae: Xylocopinae): A preferred strategy or queen coercion? *Aust J Entomol* **36**, 391-394 (DOI:101111/j1440-60551997tb01491x)

Camillo E, Garofalo CA 1982 On the bionomics of *Xylocopa frontalis* (Olivier) and *Xylocopa grisescens* (Lepeletier) in Southern Brasil I - nest construction and biological cycle *Rev Bras Biol* **42**, 571-582

Camillo E, Garofalo CA 1989 Social organization in reactivated nests of three species of *Xylocopa* (Hymenoptera, Anthophoridae) in southeastern Brasil *Ins Soc* **36**, 92-105

Chenoweth L, Schwarz MP 2007 Social biology of two Malagasy *Halterapis*: evidence that eusociality is plesiomorphic for an ancient allodapine lineage *Ann Entomol Soc Amer* **100**, 311-319

Daly HV 1988 *Bees of the new genus* Ctenoceratina *in Africa south of the Sahara (Hymenoptera: Apoidea)* University of California Press; Los Angeles, CA; ix+69 pp

Daly HV, Michener CD, Moure JS, Sakagami SF 1987 The relictual bee genus *Manuelia* and its relation to other Xylocopinae (Hymenoptera: Apoidea) *Pan-Pac Entomol* **63**, 102-124

Flores-Prado L, Chiappa E, Niemeyer HM 2008 Nesting biology, life cycle, and interactions between females of *Manuelia postica*, a solitary species of the Xylocopinae (Hymenoptera: Apidae) *New Zeal J Zool* **35**, 93-102 (DOI 0301–4223/08/3501–93)

Gerling D, Hurd PD jr, Hefetz A 1981 In-nest behaviour of the carpenter bee *Xylocopa* *pubescens* Spinola (Hymenoptera: Anthophoridae) *J Kansas Entomol Soc* **54**, 209-218

Gerling D, Hurd PD jr, Hefetz A 1983 Comparative behavioural biology of two Middle East species of carpenter bees (*Xylocopa* Latreille) (Hymenoptera: Apoidea) *Smithonian Contributions to Zoology* **369** pp1-33

Hogendoorn K, Watiniasih NL, Schwarz MP 2001 Extended alloparental care in the almost solitary bee *Exoneurella eremophila* *Behav Ecol Sociobiol* **50**, 275-282 (DOI 101007/s002650100357)

Hurst PS 2001 *Social biology of* Exoneurella tridentata*, an allodapine bee with morphological castes and perennial colonies* PhD thesis Flinders Univ Adelaide Aust

Joyce NC, Schwarz MP 2006 Sociality in the Australian allodapine bee *Brevineura elongata*: small colony sizes despite large benefits to group living *J Ins Behav* **19**, 45-61 (DOI 101007/s10905-005-9004-1)

Kislow CJ 1976 *The comparative biology of two species of small carpenter bees,* Ceratina strenua *F Smith and* C calcarata *Robertson* Ph D dissertation, University Of Georgia, Athens, Georgia, USA; III + 221 pp

Michener CD 1964 The bionomics of *Exoneurella*, a solitary relative of *Exoneura* *Pac Insects* **6**, 411-426

Michener CD 1971 Biologies of African allodapine bees (Hymenoptera, Xylocopinae) *Bull Amer Mus Nat Hist* **145**, 219-302

Neville T, Schwarz MP, Tierney S 1998 Biology of a weakly social bee *Exoneura* (*Exoneurella) setosa* (Hymenoptera: Apidae) and implications for social evolution in Australian allodapine bees *Aust J Zool* **46**, 221-234 (DOI:101071/ZO98002)

Rehan SM, Richards MH, Schwarz MP 2009 Evidence of social nesting in the *Ceratina* of Borneo *J Kans Entomol Soc* **82**, 194-209

Rehan SM, Richards MH, Schwarz MP 2010 Social polymorphism in the Australian small carpenter bee, *Ceratina (Neoceratina) australensis* *Ins Soc* **57**, 403-412 (DOI 101007/s00040-010-0097-y)

Rehan SM, Richards MH 2010 Nesting biology and subsociality of *Ceratina calcarata* (Hymenoptera: Apidae) *Can Entomol* **142**, 65-74 (DOI 104039/n09-056)

Sakagami SF, Maeta Y 1977 Some presumably presocial habits of Japanese *Ceratina* bees, with notes on various social types in Hymenoptera *Ins Soc* **24**, 319-343

Sakagami SF, Maeta Y 1984 Multifemale nests and rudimentary castes in the normally solitary bee *Ceratina japonica* (Hymenoptera: Xylocopinae) *Journal of the Kansas Entomological Society* 57(4):639-656

Sakagami SF, Maeta Y 1987 Multifemale nests and rudimentary castes of an almost solitary bee *Ceratina flavipes*, with additional observations on multifemale nests of *Ceratina japonica* (Hymenoptera, Apoidea) *Kontyu* **55**, 391-409

Sakagami SFm Maeta Y 1989 Compatibility and incompatibility of solitary life with eusociality in two normally solitary bees *Ceratina japonica* and *Ceratina okinawana* (Hymenoptera, Apoidea), with notes on the incipient phase of eusociality *Jap J Entomol* **57**, 417-739

Sakagami SF, Maeta Y 1995 Task allocation in artificially induced colonies of a basically solitary bee *Ceratina (Ceratinidia) okinawana*, with a comparison of sociality between *Ceratina* and *Xylocopa* (Hymenoptera, Anthophoridae, Xylocopinae) *Jap J Ecol* **63**, 115-150

Schwarz MP 1986 Persistent multi-female nests in an Australian allodapine bee, *Exoneura bicolor Insectes Soc* 33: 258-277

Schwarz MP, Lowe RMm Lefevere KS 1996 Kin association in the allodapine bee Exoneura richardsoni Rayment (Hymenoptera: Apidae) *Aust J Entomol* 35: 65-71

Schwarz MP, Tierney SM, Zammit J, Schwarz PM, Fuller S 2005 Social and nesting biology of a Malagasy species of *Halterapis*: implications for understanding social evolution in the allodapine bees *Ann Entomol Soc Amer* 98 : 126-133

Smith JA, Schwarz MP 2006Sociality in a Malagasy allodapine bee, *Macrogalea antanosy*, and the impacts of the facultative social parasite, *Macrogalea maizina* *Insectes Sociaux* 53(1) : 101-107 DOI:101007/s00040-005-0842-9

Steen Z 2000 *Social behaviour in endemic Australian carpenter bees* PhD dissertation, Flinders University, Adelaide, Australia 211 pp

Tierney S, Schwarz MP, Adams M 1997 Social behaviour in an Australian allodapine bee *Exoneura (Brevineura) xanthoclypeata* Australian Journal of Zoology 45(4) : 385-398 DOI:101071/ZO97022

Tierney SM, Schwarz MP, Neville T, Schwarz PM 2002 Sociality in the phylogenetically basal allodapine bee genus *Macrogalea* (Apidae: Xylocopinae): implications for social evolution in the tribe Allodapini *Biological Journal of the Linnean Society* 76(2) : 211-224 DOI:101111/j1095-83122002tb02083x

Vicidomini S 1997 Biologia di *Xylocopa* (*Xylocopa*) *violacea* (L.,1758) (Hymenoptera: Apidae): trofallassi! *Boll Mus civ nat Verona* **21**, 341-349

Watmough RH 1983 Mortality, sex ratio and fecundity in natural population of large carpenter bees (Xylocopa spp.) *J Anim Ecol*, 52: 111-125
